# Bovine telomere dynamics and the association between telomere length and productive lifespan

**DOI:** 10.1038/s41598-018-31185-z

**Published:** 2018-08-24

**Authors:** Luise A. Seeker, Joanna J. Ilska, Androniki Psifidi, Rachael V. Wilbourn, Sarah L. Underwood, Jennifer Fairlie, Rebecca Holland, Hannah Froy, Eliane Salvo-Chirnside, Ainsley Bagnall, Bruce Whitelaw, Mike P. Coffey, Daniel H. Nussey, Georgios Banos

**Affiliations:** 1 Animal & Veterinary Sciences Group, SRUC, Roslin Institute Building, Easter Bush, Midlothian, UK; 2 The Roslin Institute and Royal (Dick) School of Veterinary Studies, University of Edinburgh, Easter Bush, Midlothian, UK; 3 Queen Mother Hospital for Animals, Royal Veterinary College, University of London, Hatfield, UK; 4 Institute of Evolutionary Biology, School of Biological Sciences, University of Edinburgh, Edinburgh, Midlothian UK; 5 Centre for Systems Biology, University of Edinburgh, Edinburgh, Midlothian UK; 6 SRUC Crichton Royal Farm, Glencaple Road, Dumfries, UK

## Abstract

Average telomere length (TL) in blood cells has been shown to decline with age in a range of vertebrate species, and there is evidence that TL is a heritable trait associated with late-life health and mortality in humans. In non-human mammals, few studies to date have examined lifelong telomere dynamics and no study has estimated the heritability of TL, despite these being important steps towards assessing the potential of TL as a biomarker of productive lifespan and health in livestock species. Here we measured relative leukocyte TL (RLTL) in 1,328 samples from 308 Holstein Friesian dairy cows and in 284 samples from 38 female calves. We found that RLTL declines after birth but remains relatively stable in adult life. We also calculated the first heritability estimates of RLTL in a livestock species which were 0.38 (SE = 0.03) and 0.32 (SE = 0.08) for the cow and the calf dataset, respectively. RLTL measured at the ages of one and five years were positively correlated with productive lifespan (p < 0.05). We conclude that bovine RLTL is a heritable trait, and its association with productive lifespan may be used in breeding programmes aiming to enhance cow longevity.

## Introduction

Telomeres are structures at the ends of linear chromosomes that consist of repetitive DNA nucleotides and attached proteins of the shelterin complex^1,2^. They are crucial for chromosomal integrity and pairing of homologous chromosomes during meiosis^3,4^. In most cultured cells telomeres shorten with every cell division^5^ due to the end replication problem^6,7^. When telomeres become critically short and repair mechanisms are not activated, normal somatic cells enter apoptosis or a state called replicative senescence where they are unable to divide further^8^. Other cell types such as cancer cells^9^, embryonic tissue cells^10,11^, stem cells including hematopoietic stem cells^12^, and lymphocytes^12^ have been shown to express the reverse transcriptase telomerase^13^ that can replenish telomere length and elongate their replicative lifespan^14^ or even immortalise them^15^. However, in most cases, telomere shortening is a hallmark of cellular ageing and also seems to be associated with organismal ageing^16^. Most young individuals have longer telomeres than old individuals of the same species^17–20^, although among species TL dynamics with age vary considerably. In humans, telomere attrition with age is usually described to follow a general pattern with three stages: (1) fast telomere attrition in early life, (2) slower attrition or plateau in young adulthood and middle age, and (3) rapid depletion at older ages^21,22^. Rapid telomere attrition during early life has been observed in a wide range of species, including Soay sheep^23^, baboons^24^, European shags^25^ and wandering albatrosses^25^. However, the pattern of change in TL with age during adult life seems to vary. Adélie penguins, common terns, tree swallows, zebra finches and great frigatebirds show continuing telomere attrition at adult age^26,27^, whilst TL remains stable in adult European shags and wandering albatrosses^25^, and it actually increases in adult edible dormice^28^.

Average TL, measured in leukocytes in mammals and erythrocytes in non-mammalian vertebrates, has emerged as a potentially important biomarker of health and ageing across disciplines including epidemiology, biomedicine, ecology and animal welfare^29–36^. There is mounting evidence from across a range of species that blood cell TL is both heritable and predictive of subsequent health and mortality risk^37–39^. Heritability estimates of TL have been calculated using parent-offspring regressions, correlations between twins or pedigree-based ‘animal’ models for a variety of species including humans, sand lizards and a range of bird species. Most studies suggest that variance in TL is under some degree of genetic control^37^.

Short telomeres have been shown to be associated with higher mortality in species such as zebra finches^40^, semi-feral Soay sheep^23^ and humans^38,41^. However, other studies found no relationship between TL and lifespan^42,43^. A meta-analysis of human studies found evidence that the association between TL and mortality risk was only present at younger adult ages^38^, whilst a meta-analysis of non-human vertebrate studies found a significant overall association between TL and survival despite considerable variation among studies^39^.

Interest in the potential application of TL as a biomarker of health and welfare within the livestock industry is growing^29,44^. In particular, the improvement of so-called ‘functional longevity’ (which is productive lifespan corrected for milk yield) is currently a chief breeding goal in dairy cattle, because it would reduce the requirement for replacement heifers, minimise waste, improve animal welfare, and decrease greenhouse gas emissions and farming costs^45^. Improving functional longevity is difficult to achieve with conventional selective breeding for several reasons: phenotypes are recorded at the end of life which causes long generation intervals and slows genetic progress^46^; the recording of specific phenotypes such as reasons for culling is laborious and often not practical on a busy commercial farm; functional longevity in cattle is known to have a low heritability of approximately 0.01–0.06^47^. Therefore, an early life biomarker that is heritable and correlated with functional longevity would benefit the dairy industry tremendously, as it would enable the selection of animals in early life based on the biomarker measurement, long before they express the phenotype of interest (productive lifespan).

Currently, very little is known about TL dynamics across different ages in cattle. Telomere length in bovine embryos may be adjusted by telomerase expression at the transition from morula to blastocyst stage^48^ but literature on the post-natal TL profile is scarce. In two cross-sectional studies, young cattle were found to have longer telomeres than older animals suggesting that bovine telomeres might shorten with age^44,49^. Based on those studies it can be hypothesised that cattle would follow a human-like TL dynamics with age where telomeres shorten quickly after birth and at a slower pace thereafter.

As mentioned, for TL to be a useful biomarker for selective breeding purposes, it must be heritable. Heritability estimates for TL in non-human mammals outside the laboratory are so far missing from the literature. However, based on detection of low to moderate heritability of TL in humans and wild bird studies and the fact that domestic cattle live in a more controlled environment than either of the former species, we hypothesise that RLTL should be at least moderately heritable in cattle as well.

Crucially, the relationship between RLTL at different life stages and productive or functional longevity in dairy cattle has not been explored. Obviously, productive lifespan of dairy cows differs from longevity measurements in humans and wild animals because most cows are culled by humans. However, cows are not randomly chosen for culling, which is usually based on poor fertility or health. The only study that has addressed the association of TL with productivity in dairy cattle so far indeed found suggestive evidence that animals with short telomeres were more likely to be culled within one year of measurement^44^. However, the hypothesis that TL measured during early life in dairy cattle is predictive of future productive longevity remains to be tested.

In the present study, we repeatedly measured individual RLTL at different life stages in a large, well-monitored and pedigreed population of Holstein Friesian dairy cattle^50^. Animals were kept at the Crichton Royal research farm in Dumfries (Scotland). They represented two genetic lines (selected for high milk fat and protein yield (S) vs. control (C)) that were randomly allocated to two different diets (High forage (HF) vs. low forage (LF)) which allowed us to investigate the effect of genetic and nutritional factors on RLTL.

Our objectives were to test the following hypotheses (i) RLTL shortens with age in dairy cattle and telomere attrition is fastest shortly after birth, (ii) factors other than age such as season of birth, genetic background and diet also affect RLTL, (iii) RLTL is a heritable trait in dairy cattle and (iv) RLTL in early life is predictive of productive lifespan.

## Results

Table 1 shows the number of RLTL measurements per animal available in the cow and the calf dataset, while Tables S1 and S2 summarise other relevant information.

**Table 1:** Characterisation of the cow and the calf datasets, showing the number of animals and the number of RLTL measurements per animal (1–9).

Dataset	Total number of animals	Total number of RLTL measurements	Number of RLTL measurements per animal								
			1	2	3	4	5	6	7	8	9
Cow dataset	308	1,328	3	28	38	106	83	35	14	1	0
Calf dataset	38	284	0	0	1	2	3	5	4	7	16

All animals had an early life sample taken within two weeks of birth. Subsequent samples were taken approximately yearly and monthly for the cow and the calf datasets, respectively.

### RLTL changes with age at the population level

**Figure 1:**
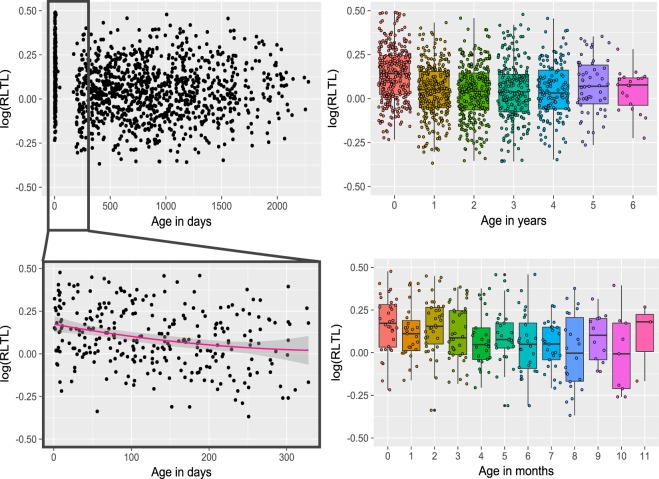
Impact of age on relative leukocyte telomere length (RLTL). Top row: Cow dataset. Log transformed RLTL over age in days (left panel) and years (right panel). Bottom row: Calf data set. Log transformed RLTL over age in days (left panel) and months (right panel). In the bottom left panel a quadratic function of age is visualised.

We observed a considerable decline in RLTL within the first year of life (estimate = −0.11, SE = 0.007, p < 0.001, Cohen’s D = −0.44) with the most rapid telomere depletion shortly after birth (Fig. 1). There was little visual evidence of a systematic change in RLTL with increasing age in animals older than one year (Fig. 1), which is consistent with the finding that modelling age as a two level factor (younger vs. older than one month of age) fitted the cow dataset best (based on AIC; Table 2). Within age group, RLTL was highly variable (CV = 0.16 and 0.18 for the cow and calf datasets, respectively). In fact, the distribution of measurements of neonates in the cow dataset overlapped that of the oldest animals (Fig. 1).

**Table 2:** Comparison of models with different functions of age.

Function of age	AIC	Delta AIC
**Cow dataset**		
/	−1431.4	193.7
linear	−1508.2	116.9
quadratic	−1575.4	49.7
cubic	−1598.6	26.5
quartic	−1605.0	20.1
age as 2-level factor	−1625.1	0
**Calf dataset**		
/	−255.89	20.74
Linear	−273.7	2.93
Quadratic	−276.63	0
Cubic	−277.64	−1.01
Quartic	−275.85	0.78
Age as 2-level factor	−276.92	−0.29

Delta AIC values are expressed in relation to the best fitting model (age as a 2-level factor and quadratic in cow and calf datasets, respectively). A delta AIC of at least 2 units corresponds to a significant difference between models (p ~ 0.05). Within two units the simper model is preferred over the more complicated one.

The cow and the calf datasets complement each other nicely in the present study. While the cow dataset provides information about RLTL throughout productive life, it does not include measurements between two and six months of age. Therefore, although we see a difference in average RLTL between neonates and older animals, we cannot be certain about the dynamics of the decline within the first few months of life. This gap is filled by the calf dataset which provides repeated measurements in early life and shows that RLTL declines with age at a slightly faster rate shortly after birth than closer to the age of one year. In the calf dataset this dynamic is best captured by a quadratic function of age or by modelling age as a two level factor (younger vs. older than four months) (Table 2), Within two AIC units the simpler model is preferred over the more complicated one^51,52^. We decided to include a quadratic function of age in the final model for the analysis of the calf dataset.

### Factors affecting RLTL independently of age

The final model for the analysis of the cow dataset contained the animal identity as a random effect and the following significant fixed effects: age as a two-level factor (younger vs. older than one month; estimate difference = −0.11, SE = 0.007, p < 0.001), birth year of cow (p = 0.004), qPCR plate (p < 0.001) and qPCR row (p < 0.001) (Supplementary Table S5). For the analysis of the calf dataset, the final model included the animal identity as a random effect, a quadratic function of age (linear estimate = −0.606, SE = 0.138, p < 0.001 and quadratic estimate = 0.294, SE = 0.13, p = 0.031), birth weight (p = 0.04) and birth season (p = 0.04) of the calf, qPCR plate (p < 0.001), and qPCR row (p < 0.01) as fixed effects. Heavier calves had marginally shorter RLTL at birth (estimate difference = −0.005, SE = 0.002, p = 0.045) and calves born over the winter months (October to March) had longer telomeres (estimate difference = 0.069, SE = 0.033, p = 0.041). Supplementary Tables S5 and S6 and Supplementary Figs S1–S4 summarise all significant effects.

Interestingly, neither in the cow dataset nor in the calf dataset was the genetic group of the animal significantly associated with RLTL. Also, there was no statistically significant relationship between the feed group and RLTL in the cow dataset.

### Estimation of genetic and environmental variance components

The animal effect in the model included pedigree information, which enabled the estimation of the genetic variance for RLTL. A permanent environment random effect was also fitted to account for common non-genetic variance of repeated measures within individual animals. Estimates of variance components and genetic parameters are shown in Table 3. The variance due to permanent environment was practically zero. Therefore, the permanent environment effect on RLTL in our datasets is negligible. Heritability estimates of RLTL were 0.38 (SE = 0.03) and 0.32 (SE = 0.08) for the cow and the calf datasets, respectively.

**Table 3:** Variance components and genetic parameters for RLTL in the cow and the calf dataset.

Dataset	Phenotypic variance	Genetic variance	Permanent environment variance	Residual variance	Permanent environment	Repeatability	Heritability
cow	0.0037 (1.9 × 10^−4^)	0.0014 (0.0002)	1.8 × 10^−10^ (8.1 × 10^−12^)	0.0023 (1.03 × 10^−4^)	0.0 (0.0)	0.3832 (0.03)	0.3832 (0.03)
calf	0.0047 (5.6 × 10^−4^)	0.0015 (0.0005)	2.5 × 10^−9^ (2.3 × 10^−10^)	0.0032 (2.97 × 10^−4^)	0.0 (0.0)	0.3231 (0.08)	0.3231 (0.08)

Estimates are followed by standard errors in brackets.

### Relationship between RLTL and productive lifespan

Livestock animals are usually culled at the end of their life. Therefore, their longevity measures have to be defined differently than for members of natural populations. We define “productive lifespan” as the survival time from birth to culling measured in days. “Functional longevity” is defined as productive lifespan corrected for milk yield and is a measure that is often used in genetic studies of dairy cattle. A total of 244 out of 308 animals in the cow dataset were culled thereby providing phenotypes for productive lifespan measurements. For most of them (220 out of 244) a precise culling reason was recorded (Supplementary Table S7). The majority of animals were culled involuntarily, particularly due to fertility problems, mastitis or lameness. Productive lifespan ranged from 17 to 2,823 days (mean = 1,477 days) (Fig. S6), which means that on average cows were culled during their third lactation. There was no difference in productive lifespan between the two genetic lines (Welch two sample t-test: t = −0.8954, df = 242, p-value = 0.3714) and between the two feed groups (Welch two sample t-test: t = −1.1235, df = 170, p-value = 0.2628).

In the present study we were interested in the potential use of RLTL as an early life trait associated with productive lifespan. Therefore, the subsequent analyses were focused on RLTL measurements at birth and at the age of one year. We defined functional longevity as productive lifespan adjusted for milk yield. For this reason, we fitted a linear model with productive lifespan as the response variable and the average milk yield over the first four lactations as a covariate. Subsequently, we fitted the animal’s RLTL measurement at birth or at the age of one year (separate analyses) as a second covariate to the model to assess its relationship with functional longevity. In either case, RLTL had been first pre-adjusted for qPCR plate and row by fitting those factors as fixed effects and using the residuals of the model for further analysis. Because animals were required to have both productive lifespan and milk yield records over the first four lactations available, these analyses were based on 143 and 136 animals with RLTL measurements at birth and one year of age, respectively. Milk yield significantly affected lifespan of those animals (estimate = 81.13, SE = 13.92, p < 0.001) but RLTL neither at birth (estimate = −11.10, SE = 329.77, p = 0.973) nor at the age of one year (estimate = 355.91, SE = 394.42, p = 0.368) had a significant effect.

**Figure 2:**
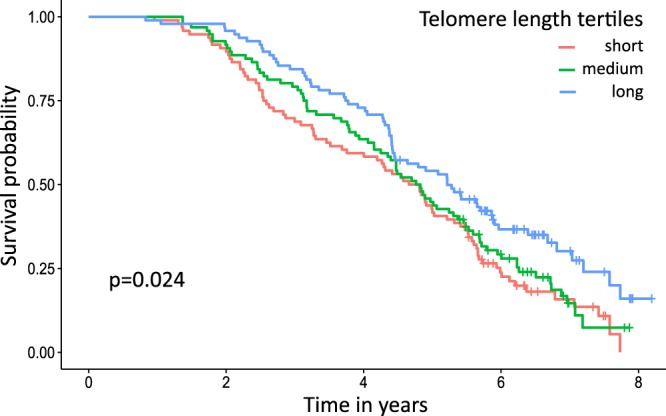
Kaplan-Meier plot for survival probability in relation to telomere length tertile at the age of 1 year.

However, the analysis of functional longevity excluded animals that might be of particular interest: animals that died at a young age were excluded for not having productivity records; also animals that were still alive and belonged to the group of animals with the best productive lifespan were excluded, because they did not have a productive lifespan measurement yet. Therefore, we tested the phenotypic correlation between productive lifespan and functional longevity (r = 0.9, p < 0.001, see Supplementary Fig. S5) and found that productive lifespan may be used as a proxy trait for functional longevity in the present study. This allowed us to include all animals that died before generating productivity measurements. Also, we considered a Cox proportional hazard analysis that enabled us to include all animals that were still alive by including a censoring group in the analysis. With those measures we increased our sample size from 143 to 305 RLTL measurements at birth and from 136 to 284 measurements at the age of one year. With the Cox proportional hazard analyses we investigated the survival time in years after sampling. We found that RLTL at birth was not a predictor of survival (p = 0.234), but there was a significant linear relationship between RLTL at the age of one year and subsequent survival (estimate = −1.974, SE = 0.708, p = 0.005). The estimate is negative, because it describes the relation between RLTL and mortality which is the opposite of survival. RLTL at subsequent ages were also tested. RLTL at the ages of 2–4 years did not relate to productive lifespan, but RLTL at the age of 5 years was correlated with subsequent survival (N = 53, estimate = −3.267, SE = 1.346, p = 0.015) (Table 4). We recognise the presence of multiple statistical tests in this part of analysis. Tests were not completely independent since both RLTL measurements and survival at different ages are correlated traits. Nevertheless, we implemented the relatively strict Holm-Borferroni correction^53^ according to which the most significant result in year one maintained its statistical significance but the year five result narrowly lost it. Because of the lack of test independence these corrections should be viewed with caution. In any case, our results support the presence of a significant relationship between an early RTL measurement and subsequent survival. The relationship between RLTL at the age of one year (grouped into tertiles) and productive lifespan is visualised in Fig. 2.

**Table 4:** Relationship of RLTL measured at different ages with subsequent survival.

RTL at age in years	N	Beta coefficient (SE)	Hazard Ratio (95% CI)	Wald statistic	α-value after Holm-Bonferroni correction	p-value
0	305	0.768 (0.661)	2.155 (0.591–7.866)	1.35	/	0.245
1	284	−1.974 (0.708)	0.139 (0.035–0.556)	7.78	0.0083	0.005
2	261	0.763 (0.682)	2.144 (0.563–8.16)	1.25	/	0.264
3	220	−1.285 (0.744)	0.277 (0.064–1.188)	2.99	/	0.084
4	208	−1.147 (0.763)	0.318 (0.071–1.416)	2.26	/	0.133
5	53	−3.267 (1.346)	0.038 (0.003–0.533)	5.89	0.01	0.015

CI: Confidence interval.

## Discussion

This is the first large-scale longitudinal study of RLTL dynamics conducted in farm animals and one of the few lifelong studies reported in any species. The combination of calf and cow datasets provided valuable information allowing us to better understand telomere dynamics in early life and throughout the animal’s productive life, respectively. Our results show that average RLTL declines within the first year of life but remains relatively stable thereafter. Telomere attrition shortly after birth has been reported in other mammals such as humans^21,22,54^, baboons^24^ and Soay sheep^23^ as well as some birds such as Seychelles warblers^17^, European starlings^55^, corvids^20^ and Jackdaws^56^, and is probably due to the high number of cell divisions that are necessary for the body to grow quickly during that time. Also, postnatal maturation of the immune system and the sudden challenge to fight off pathogens after birth might cause quick telomere depletion within the first months. Varying results have been reported about telomere dynamics in adult life in different species. While some studies in humans, badgers and birds show a linear telomere decline throughout adult life^17,18,20,21,54^, a study on the semi-feral Soay sheep reported more complex dynamics: telomeres shortened within the first four months of life, then lengthened until the age of 5 years and shortened afterwards again^23^. Other studies in mammals and birds report similar results to ours with no relation between age and TL after the initial decline early in life^24,25,57^. A particularly interesting telomere changing pattern has been observed in edible dormice: after the initial decline in early age, telomeres elongate considerably in adult life^28^. Those are a few examples that illustrate that telomere dynamics across life stages vary among species. Our results do not support former studies on TL in cattle that reported an age-dependent decline in adult life^44,49^. Former studies may have found TL attrition with age, because they included older animals in their analyses (maximum age of 13 or 14 years^44,49^ compared to six years in the present study). It is possible that bovine RLTL dynamics are similar to those observed in humans with fast telomere attrition in early life, a plateau in younger adulthood and possibly a second decline later in life that we failed to detect in the present study because we did not test animals that were older than six years. Another explanation may be that cattle indeed maintain their telomere length through the expression of telomerase in hematopoietic stem cells or lymphocytes. The function and role of telomerase were beyond of the scope of the present study but need to be addressed in future research.

To our knowledge we report in the present study the first heritability estimates for RLTL in a livestock species. Presence of significant genetic variation and heritability of bovine RLTL suggests that the trait may be altered with selective breeding; heritability estimates of RLTL in cattle were relatively high (0.31–0.39) and within the range of estimates reported for other species such as humans (h^2^ = 0.28–0.76)^41,58–64^ and birds (h^2^ = 0–0.999)^65–69^. The human studies benefitted in comparison to the bird studies from larger sample sizes^37^.

One factor that might affect our heritability estimates of RLTL is the potential presence of interstitial telomeres in the cattle genome. Interstitial telomeres are telomeric sequences that are not located at the ends of chromosomes but between centromeres and telomeres^70–75^. Interstitial telomeres are believed to be constant in their amount between tissues and also to remain constant in their length with ageing. Therefore, their presence could lead to an over-estimation of the heritability of RLTL. However, little is known about the presence of interstitial telomeres in the cattle genome to date. For future studies it would be interesting to measure bovine TL using a method that exclusively measures telomeric sequences at the ends of chromosomes (in gel TRF^76^) which will provide more accurate heritability estimates if interstitial telomere are present in the cattle genome and if their amount varies between individuals.

The two genetic groups in our study population, which had been selected to differ considerably in milk production, did not differ in their mean RLTL. Based on these results an unfavourable genetic correlation between TL and productivity is highly unlikely which is desirable because it implies that TL may be altered with genetic selection within a breeding programme without reducing the milk yield of cows.

We found that feed group of the animals did neither affect RLTL nor the productive lifespan. It has been shown that calorie restriction improves longevity in many species^77^ and also that diet affects TL in humans^78–80^. However, the human diet varies a lot more between individuals and there is a greater potential for truly unhealthy and harmful eating habits in humans than in cattle. For example, Nettleton *et al*. (2008) found a negative effect of the consumption of processed meat on TL, while Cassidy *et al*. (2010) found a positive effect of dietary fibre intake^78,80^. While the two feed groups in the present study differ in energy content as defined in separate feed experiments on this population^81–83^, they can both be considered as healthy and fibre rich.

In the analysis of the calf dataset we found a significant relationship between birth weight and RLTL, with heavier calves having shorter telomeres. This is in accordance with the theory that faster body growth requires more cell divisions and, therefore, bigger sized individuals of the same species have shorter telomeres^84^. However, it is in contrast to former studies in humans that found no relation of birth weight and TL^85,86^ or longer telomeres in bigger and heavier babies^87^. In our study, birth weight might be confounded with gestation length. Heavier calves may have been born past their due date and their having shorter telomeres could be a reflection of fast age-dependent telomere depletion before birth which usually happens during the first days of life.

It is not clear if the birth year effect on RLTL in the cow dataset reflects random noise associated with measurement error or true biology. Year-dependent changes in weather, food quality or other environmental factors may affect RLTL measurements systematically. Birth cohort effects on RLTL have been observed before in wild Soay sheep^23^ which could support the presence of a true biological effect of birth year on the animals’ RLTL. However, in contrast to Soay sheep, cattle in our study were born and lived in a controlled environment which makes biological cohort effects less likely. The observation of a potential season effect on RLTL of calves has not been reported before and might be explained by different pregnancy conditions either due to more daylight over the summer months or due to more fresh feed available during the grazing period. Stress^88,89^ and nutrition^90^ during pregnancy have been reported to affect TL in humans and rats respectively. Also, experimentally elevated stress hormones during pregnancy and early post-natal life conditions have been associated with shorter telomeres or faster telomere shortening in birds^56,66,91,92^. Besides those studies, little is known about factors during pregnancy and early life that affect the offspring’s TL and more specifically designed studies are required to gain a better understanding. For future studies on RLTL it would be interesting to investigate the possible effect of the dam’s feed group on the calves’ RLTL, which may be associated with the birth season effect. Also, the inclusion of a possible maternal effect in future analyses would be interesting.

No significant association of RLTL either at birth or at one year of age with functional longevity (productive lifespan adjusted for milk yield) was found in the present study. The sample size might have been too small to detect such an effect. However, when we included more animals by investigating productive lifespan as a proxy for functional longevity and by introducing a censoring group in the Cox proportional hazard analysis to include animals that were still alive, we found that animals with the longest telomeres at the ages of one and five years lived significantly longer (p < 0.05). While the result for RLTL at one year remained statistically significant even after employing a Holm-Bonferroni correction^53^ to account for multiple testing, the result for RLTL at the age of five years became non-significant, indicating that it may have been caused by a statistical artefact. There was no significant signal for RLTL at birth and other ages. Former studies on the potential value of telomeres as a biomarker for lifespan in humans and laboratory animals have shown mixed results: whilst some studies found a relationship between short telomeres and reduced lifespan or the early onset of age related symptoms and diseases^93–95^ other studies could not find a relationship^42,43,96,97^. Two meta-analyses on humans and non-human vertebrates suggest an overall significant positive relationship between TL and lifespan^38,39^. In dairy cattle, the only other study on RLTL in relation with productive lifespan so far showed a significant but weak association^44^. Cows with short telomeres were more likely to be culled within the next year regardless of their age at sampling. It is not clear why we detected an association between TL and productive lifespan at the ages of one (and five years) but not at other ages.

The present study was based on average TL measured with a high-throughput method applicable to a large sample size. Other TL measurement techniques^98–100^ that allow not only the analysis of average TL but also of the variance and range of TL in relation to productive lifespan would be useful in future studies. Those studies might find strong relationships between telomere length and productive lifespan, because it is likely that the accumulation of very short telomeres within a tissue is problematic for the organism^101^. However, the current state of development of such alternative TL measurement techniques does not allow high-throughput process in a large-scale study like the present one. When these techniques are further developed, future studies can shed more light into TL dynamics, as discussed above. Future studies will also need to investigate the relationship between telomerase expression and productive lifespan in dairy cattle.

In order to clarify if RLTL could be used as a biomarker for breeding for improved productive lifespan it would be interesting to investigate the genetic correlation between RLTL and productive lifespan. Genetic correlations between two traits estimated with bivariate analyses, in contrast to phenotypic correlations estimated in the present study, will not be affected by environment effects and might therefore deliver different results.

There is growing interest in the change in TL rather than single measurements at specific ages in association with lifespan. Several studies in birds have indeed shown that the rate of change in TL might be more strongly associated with lifespan than mean TL measurements^17,20,55,56^. This type of association in dairy cattle warrants further investigation.

## Materials and Methods

### Study system and sample selection

Holstein Friesian dairy cattle used in the present study belong to the herd kept at the Crichton Royal research farm in Dumfries (Scotland) where they are closely monitored and repeatedly sampled for a broad range of scientific studies, including feeding, greenhouse gas emission and genetics experiments. Since 1976 two distinct genetic lines are maintained at the farm: a selection (S) line and a control (C) line^50^. Cows of the S line are amongst the highest yielding dairy cows in the UK, due to a careful selection of breeding bulls with the highest genetic merit for total milk, fat and protein production internationally. Cows of the C line have been deliberately kept at an average UK performance level. Animals of the two genetic lines are randomly allocated to two different feeding groups after first calving (calf birth): a high forage (HF) and a low forage (LF) diet group. While the HF group is turned out to the fields during the summer months, the LF group is housed continuously. The LF diet is based on by-products of plants farmed for human consumption and is richer in energy and protein content than the HF diet which is based on farm grown fibre-rich feed. Feeding affects the metabolism and milk yield of a dairy cow: a cow of the S line has a targeted milk yield of 7,500 litres per year when managed on a HF diet and of 13,000 litres per year when managed on a LF diet. The farm keeps 50 milking cows of each genetic and feeding group at all times which makes it ca. 200 milking animals in total. The monitoring of the animals includes regular measurement of milk yield, body condition score, body weight, health events, feed intake, fertility measurements, calving records, productive lifespan measures, reasons for culling etc. Cows stay in the experiment for three lactations or five years. Afterwards, they are transferred to the Acrehead Dairy Unit where they are still used commercially. While the feeding experiment stops at that point and animals are monitored less intensively, regular blood samples and basic records are still collected until culling.

### Blood sampling and sample selection

A blood sample archive for the cattle of the Crichton herd was started in 2004 and routine sampling protocols include initial blood sampling within the first two weeks of life and an annual sampling of all animals in spring. When possible, an additional sample is taken close to the animal’s planned culling (removal from the herd). Whole blood samples are taken by venepuncture (V. jugularis for calves and V. coccygealis for adults) and EDTA is used as an anticoagulant. Samples are stored at −30 °C. In the present study we used these blood samples to extract DNA and measure TL of individual animals. In the first instance, we selected 1,336 blood samples from 308 individual cows taken at different ages (cow dataset). Since few samples were collected on these animals within the first year of life, we collected an additional 284 blood samples from 38 female calves over the course of their first year of life, at approximately monthly intervals (calf dataset).

### Ethics statement

Blood sampling was approved by the SRUC Animal Experimentation Committee and conducted in accordance with UK Home Office regulations (UK Home Office Project License Number: PPL 60/4278 Dairy Systems, Environment and Nutrition).

### DNA extraction

Leukocyte DNA was extracted using spin columns of the DNeasy Blood & Tissue kit (QIAGEN). We followed the manufacturer’s protocol for the extraction from whole blood samples with certain alterations^102^. DNA yield and purity were measured for each sample on a NanoDrop ND-1000 spectrophotometer (Thermo Scientific) with the software NanoDrop 2000 and the DNA integrity of each sample was tested with gel electrophoresis (as recommended by Kimura *et al*.^99^). We processed samples which met the following requirements: yield >20 ng/μl, 260/280 ratio >1.7 and 260/230 ratio >1.8, integrity gel score <3 (according to Seeker *et al*.^102^). We extracted DNA with acceptable quality from 1,612 out of 1,620 whole blood samples (1,328 samples belonged to the cow dataset and 284 samples to the calf dataset).

### Quantitative polymerase chain reaction (qPCR)

Average RLTL was measured by qPCR^103^ as described in Seeker *et al*.^102^. Average RLTL is expressed as the amount of telomeric DNA in relation to the amount of a reference gene that is constant in copy number. Both the telomere reaction and the reference gene reaction were performed in different wells but on the same qPCR plate (monoplex qPCR). One sample –the so called calibrator or golden sample- was repeated on each plate and included in the calculation of RLTL to correct for random measurement error and to allow a comparison of RLTL measurements from different qPCR plates. The calibrator sample was extracted using the same protocol as for the samples in the study (QIAGEN Blood & Tissue kit spin columns)^102^.

The master mixes for the telomere and the reference gene reaction contained 5 μl of LightCycler 480 SYBR Green I Master (Roche) per well. For the telomere amplification, tel 1b (CGG TTT GTT TGG GTT TGG GTT TGG GTT TGG GTT TGG GTT) and tel 2b (GGC TTG CCT TAC CCT TAC CCT TAC CCT TAC CCT TAC CCT)^104^ primers were used at a concentration of 900 nmol. They were manufactured and purified with high performance liquid chromatography by Integrated DNA Technologies (IDT, Glasgow, UK). For the reference gene amplification beta-2-microglobulin (B2M) primers (Primerdesign, accession code NM_001009284) were used at a concentration of 300 nmol^102^. Nuclease free water was added to the master mixes to allow for a final volume of 10 μl per qPCR plate well. The reference gene B2M has been previously tested against other candidate genes for a consistent copy number using the qbase+ software by Primerdesign and showed stable qPCR results^102^. This gene has been used for RLTL measurement in different ruminant species such as Soay sheep^23,105,106^, roe deer^107^ and Holstein Friesian dairy cattle^102^.

Samples were randomly allocated to a qPCR plate and well. A 96 well sample plate was loaded prior to loading the qPCR plate with the following (20 μl each): fifty-six DNA samples at a concentration of 1 ng/μl, two replicates of a calibrator at 1 ng/μl, a five step 1:4 serial dilution of golden sample DNA starting with 20 ng/μl and a negative control (nuclease free water).

A liquid handling robot (Freedom Evo-2 150; Tecan, Mannedorf, Switzerland) was used to transfer both master mixes and the contents of the sample plate in triplicates onto a 384 well plate. The robot mixed 9 μl of the respective master mix with 1 μl of the contents of the sample plate per well.

We used the following qPCR protocol on a LightCycler 480 (Roche): 15 min at 95 °C for enzyme activation followed by 50 cycles of 15 s at 95 °C (denaturation), 30 s at 58 °C (primer annealing) and 30 s at 72 °C (signal acquisition). For the production of the melting curve 1 min at 95 °C was followed by 30 s at 58 °C and a continuous increase of 0.11 °C/s to 95 °C with continuous signal acquisition. The protocol was finalised by a 10 s cool down at 40 °C.

### qPCR quality control

The software package LinReg PCR^108^ was used for a baseline-correction of the amplification curves, the calculation of the plate and reaction specific qPCR efficiency, and the calculation of Cq values. The plate and reaction specific efficiency was calculated by averaging across all well-specific efficiencies of the respective reaction (B2M or telomere reaction) in each plate, after excluding the top and bottom 5%.

The reaction specific efficiencies for all 25 qPCR plates ranged from 94.3% to 94.85% for the B2M reaction and from 91.5–95.85% for the telomere reaction.

For the calculation of Cq values, thresholds were set for all plates within the window of linearity; thresholds were 0.221 for the B2M reaction and 0.256 for the telomere reaction.

Individual samples failed the quality control if the Cq values across their triplicate varied too much (CV >5%). They also failed if the efficiency for at least one copy within the triplicate was 5% higher or lower than the respective mean plate efficiency (across the B2M or telomere reaction).

We ran two triplicates of the calibrator sample on each plate, one located in the middle of the plate and the other at its periphery. For the calibrator sample, Cq values and efficiencies had to meet our quality control criteria calculated over all six wells (both triplicates) per reaction to ensure high intra-plate repeatability.

To test the accuracy of our qPCR assay, we repeated the measurement of an identical qPCR plate four times over two days. Correlation coefficients for all possible plate combinations were high (r = 0.75–0.89) which implies that the rank order of samples stayed similar over all measurements. We fitted a mixed linear model with qPCR plate as fixed effect and sample ID as random effect and calculated a repeatability (variance due to the sample divided by total variance) of 80%.

Samples that failed the post-qPCR quality control (26 in total; 1.55%) were measured again and all passed quality control at the second attempt.

### RLTL calculation

RLTL was calculated using following formula published by Pfaffl^109^:

1 $$RLTL=\frac{{E}_{TEL}^{C{q}_{TEL(Calibrator)}\,-\,C{q}_{TEL(Sample)}}}{{E}_{B2M}^{C{q}_{B2M(Calibrator)}\,-\,C{q}_{B2M(Sample)}}}$$

where E_TEL_ and E_B2M_ are the reaction and plate specific qPCR efficiencies, Cq is the number of cycles after which the amplification curve crosses the set fluorescence threshold, Cq_TEL(Calibrator)_ and Cq_B2M(Calibrator)_ are the Cq values for the calibrator sample for the respective reaction and Cq_TEL(Sample)_ and Cq_B2M(Sample)_ are the Cq values for the respective reaction of the individual sample.

### Statistical analysis

All RLTL measurements were log transformed to achieve normal distribution (Shapiro-Wilk normality test: W = 0.9985, p-value = 0.2988 and W = 0.9949, p-value = 0.4604 for the cow and the calf datasets respectively).

We used mixed models to assess the effect of various factors (including age) on RLTL and to calculate variance components and genetic parameters. We started by fitting a basic model including the animal identity as a random effect and qPCR plate and the sample position on the qPCR plate (row) as fixed effects. Our previous work has shown that the calibrator sample is not sufficient to completely correct for the measurement error that is associated with the qPCR plate^102^ and therefore we needed to correct for it statistically. Position effects in qPCR assays have also been observed to cause measurement error in previous studies^110,111^. We found in a preliminary test with one identical sample repeated in all wells that the best way to adjust for the well effect is by fitting the row in which the sample was located in the model as a fixed effect.

Next we investigated RLTL dynamics with age by comparing models that incorporated age fitted in different ways in addition to the factors of the basic model. Linear, quadratic, cubic and quartic polynomial functions of age were fitted and assessed based on their AIC values, because models were non-nested (Table 2). Low AIC values were desirable. Within the range of two units (which is corresponding to an approximate significance of p < 0.05) the simpler model was preferred over the more complicated^51,52^. Because of a distinct difference in RLTL measurements between neonates and older animals in the cow dataset, we tested age as a factor with two levels. We computed separate models for all possible points of separation between the age groups (age in months) and then selected the age cut-off that minimised the residual sum of squares (Table S3). For consistency the same procedure was applied to the calf dataset (Table S4). Once we were satisfied with the age term in our models, other factors that might affect RLTL were tested. For the cow dataset we examined the animal’s genetic and feed group, the birth year and the birth season as fixed effects and the animal’s birth weight as covariate. We also included all possible interactions between main effects. We were specifically interested in the interaction of age with the animal’s birth weight, birth year and birth season, because we suspected that those effects might influence RLTL shortly after birth, but probably not later in life. All non-significant factors and interactions (P > 0.05) were backwards eliminated. The effect size of the age factor in the cow dataset was estimated by calculating Cohen’s D as the mean difference between factor level estimates divided by the standard deviation. The standard deviation was calculated as the product of the standard error and the square root of the sample size. For the calf dataset we examined the following fixed effects: a quadratic function of age in days, the genetic group of the animal, birth weight, birth year, and birth season. Feed group was not tested, because animals of the calf group were too young to have been allocated to a feeding group; the latter usually takes place when the cow gives birth for the first time at about two years of age. We backwards eliminated all non-significant fixed effects with a p-value greater than 0.05. For both datasets, the investigation of the best fitting function of age was repeated with all significant fixed effects in the model.

The final models were used to estimate variance components and genetic parameters based on the principle of restricted maximum likelihood. Animal pedigree information was fitted to the random animal effect enabling the estimation of genetic variance for RLTL. The pedigree included 11,003 animals spread over 27 generations. The animals in the cow dataset were offspring to 40 sires and 241 dams. The animals in the calf dataset were offspring of 7 sires and 35 dams. A random permanent environment effect was also fitted to account for repeated measures within individual animal. The sum of the genetic, permanent environment and residual variances yielded estimates of the total phenotypic variance of RLTL. The ratio of genetic to total phenotypic variance was used to estimate the narrow-sense heritability of the trait. The ratio of the sum of genetic and permanent environment to the total phenotypic variance was used to estimate the repeatability of the trait. The ASReml software^112^ was used for the estimation of variance components and calculation of their ratios.

Next, we investigated the association between RLTL and lifespan measurements. We defined “productive lifespan” as the age in days of the animal at culling and functional longevity as productive lifespan corrected for milk yield. To assess the relationship between functional longevity and RLTL we fitted linear models with productive lifespan (normal distributed, Shapiro-Wilk normality test: W = 0.9889, p = 0.31) as response variable and the average milk yield calculated over the cow’s lifetime as fixed effect. We added RLTL measurements that were pre-adjusted for qPCR plate and row as second fixed effect to the model. Because animals had to have both productive lifespan and productivity measurements to be included in the analysis, estimations were based on 143 animals for RLTL at birth and on 136 animals for RLTL at the age of one year only.

We investigated the possibility of analysing productive lifespan as a proxy for functional longevity by testing their correlation, because the analysis of the former would allow us to include animals that died before providing a productivity measurement in our calculations. Also, we introduced a censoring group to account for animals that were still alive and therefore had no productive lifespan measure yet. We used Cox proportional hazard models for the subsequent analyses. These measures increased the sample size to 305 at birth and to 53 at the age of one year. With the Cox proportional hazard analysis we tested the subsequent survival time after sampling at the ages of 0, 1, 2, 3, 4 and 5 years. RLTL measurements were pre-adjusted for qPCR plate and row. Significance of RLTL at test all test ages on productive lifespan was estimated using the Wald test. One might argue that our analysis qualifies to be considered as multiple testing which would require a Bonferroni correction. We therefore used a Holm-Bonferroni correction where the p-value associated with RLTL at the age of one year had to be smaller than 0.05/6 = 0.0083 and the p-value associated with RLTL at the age of five years had to be smaller than 0.05/5 = 0.01 to remain statistically significant^53^. To visualise the association of TL at the age of one year with survival probability we repeated the Cox proportional hazard analysis using TL tertiles at this age.

If not stated otherwise, statistical analyses were performed in R studio with R 3.1.3^113^. Mixed-effects models were implemented using the ‘lme4’ library. Cox proportional hazard analysis was conducted using the package ‘survival’. The significance threshold for all statistical analyses was set to α = 0.05.

## Electronic supplementary material

Supplementary Information

## Data Availability

All data are available at https://github.com/LASeeker/Bovine-telomere-dynamics.
