# Supplementary material for: Bovine telomere dynamics and the association between telomere length and productive lifespan
Source: Sci Rep. 2018 Aug 24;8:12748. doi: 10.1038/s41598-018-31185-z (PMC6109064; doi:10.1038/s41598-018-31185-z)
Supplement: Supplementary file 1 — Supplementary Information [file 41598_2018_31185_MOESM1_ESM.pdf]

Supplementary File 1

Bovine telomere dynamics and the association between telomere length and productive lifespan

Authors & Affiliations:

Luise A. Seeker[1,2]*, Joanna J. Ilska[1], Androniki Psifidi[2,3] , Rachael V. Wilbourn[4], Sarah L. Underwood[4], Jennifer Fairlie[4], Rebecca Holland[4], Hannah Froy[4], Eliane Salvo-Chirnside[5], Ainsley Bagnall[6], Bruce Whitelaw[2], Mike P. Coffey[1], Daniel H. Nussey[4] & Georgios Banos[1,2]

[1] Animal & Veterinary Sciences, SRUC, Roslin Institute Building, Easter Bush, Midlothian EH25 9RG

[2] The Roslin Institute and Royal (Dick) School of Veterinary Studies, University of Edinburgh, Easter Bush, Midlothian, UK

[3] Royal Veterinary College, University of London, Hatfield, UK[4] Institute of Evolutionary Biology, School of Biological Sciences, University of Edinburgh, Edinburgh, Midlothian, UK

[5] Centre for Systems Biology, University of Edinburgh, Edinburgh, Midlothian, UK

[6] SRUC Crichton Royal Farm, Glencaple Road, Dumfries, UK

*corresponding author:

Luise.seeker@ed.ac.uk

Table S1: Number of animals by genetic and feeding groups.

	Genetic select group	Genetic control group	Total
	Cow dataset		
High forage diet	57	53	110
Low forage diet	58	60	118
Not allocated to diet	20	60	80
Total	135	173	308
	Calf dataset		
Not allocated to diet	22	16	38

Table S2: Number of animals by birth year and season. The birth season reflects a difference in daylight but also management of the animals: While animals of the LF diet are housed continuously, animals of the HF diet are turned out for grazing from April to September.

	Cow dataset	Calf dataset
Birth year		
2008	25	/
2009	90	/
2010	89	/
2011	70	/
2012	28	/
2013	4	/
2014	2	36
2015	/	2
Birth season		
1 (April to September)	152	19
2 (October to March)	156	19

Table S3: Cow dataset: Comparison of models that included age as a factor with two levels (younger vs. older at the given age in months). AIC: Akaike information criterion. The smaller the AIC, the better is the model fit. Results are sorted by the AIC value (first AIC is the smallest).

Age in months	AIC	Age in months	AIC	Age in months	AIC	Age in months	AIC
1	-1625.13	20	-1495.86	36	-1445.49	52	-1436.58
3	-1624.3	21	-1491.87	40	-1445.2	51	-1436.55
4	-1624.3	22	-1491.59	41	-1445.19	59	-1436.51
5	-1624.3	23	-1483.33	42	-1442.93	66	-1436.39
6	-1624.3	24	-1482.06	43	-1441.93	54	-1436.18
2	-1623.71	25	-1475.31	44	-1441.43	53	-1436.17
7	-1615.92	26	-1474.58	45	-1440.83	63	-1436.17
8	-1591.34	27	-1469.37	47	-1439.66	58	-1436.15
9	-1574.94	28	-1467.7	48	-1439.49	60	-1436.06
10	-1554.28	30	-1462.34	46	-1439.31	61	-1436.06
11	-1554.27	29	-1461.71	69	-1437.91	55	-1436.01
12	-1535.6	31	-1457.9	49	-1437.88	56	-1436.01
13	-1525.91	32	-1453.99	68	-1437.69	57	-1435.99
14	-1521.79	33	-1451.78	50	-1437.57	64	-1435.98
15	-1511.8	34	-1449.02	70	-1437.42	62	-1435.98
16	-1500.09	39	-1446.96	71	-1437.23	75	-1435.97
18	-1498.82	35	-1446.84	72	-1437.23	65	-1435.96
17	-1498.57	38	-1446.3	73	-1437.23	67	-1435.96
19	-1496.74	37	-1445.7	74	-1437.23		

Table S4: Calf dataset: Comparison of models that included age as a factor with two levels (younger vs. older at the given age in months). AIC: Akaike information criterion. The smaller the AIC, the better is the model fit. Results are sorted by the AIC value (first AIC is the smallest).

Age in Months	AIC
4	-276.92
3	-273.976
5	-272.586
6	-269.329
2	-263.047
7	-262.209
1	-260.774
8	-257.075
9	-254.115
11	-254.007
10	-253.898

Table S5: Cow dataset. Fixed effect estimates of the final model. Age was fitted as a two level factor (younger vs. older than one month) and animal ID was fitted as random effect. Variance components are shown in table 2 of the manuscript.

	Estimate	Std. Error	df	t value	p value for factor level	p value for factor
(Intercept)	0.103676	0.028125	744.4827	3.686298	0.000244063	
age over 1 month	-0.10599	0.007469	1012.862	-14.1911	<0.0001	<0.0001
birth year 2009	0.035954	0.023252	275.7098	1.546259	0.123189057	0.004
birth year 2010	-0.02469	0.023353	279.6671	-1.0573	0.291286032	0.004
birth year 2011	-0.00911	0.024263	290.0133	-0.37558	0.707504856	0.004
birth year 2012	-0.02104	0.029774	334.91	-0.70659	0.480312438	0.004
birth year 2013	0.075361	0.063079	460.202	1.194703	0.232818364	0.004
birth year 2014	0.049842	0.094348	599.1338	0.528281	0.597499673	0.004
qPCR plate 2	0.025009	0.023093	1149.339	1.082953	0.27905642	<0.0001
qPCR plate 3	0.021179	0.023263	1157.139	0.910411	0.362795349	<0.0001
qPCR plate 4	0.008526	0.023532	1153.672	0.36229	0.717201957	<0.0001
qPCR plate 5	-0.00539	0.023228	1151.427	-0.2321	0.816504973	<0.0001
qPCR plate 6	-0.00944	0.023302	1158.579	-0.40528	0.685343015	<0.0001
qPCR plate 7	0.01214	0.023483	1147.634	0.516977	0.605272151	<0.0001
qPCR plate 8	-0.00379	0.02302	1138.496	-0.16484	0.869100473	<0.0001
qPCR plate 9	-0.00696	0.023301	1127.711	-0.29876	0.765179091	<0.0001
qPCR plate 10	0.034607	0.023038	1141.303	1.502143	0.133336876	<0.0001
qPCR plate 11	0.013568	0.023145	1156.444	0.586212	0.557847197	<0.0001
qPCR plate 12	0.036181	0.023322	1156.302	1.551387	0.121082682	<0.0001
qPCR plate 13	0.061542	0.023235	1141.643	2.648723	0.008191132	<0.0001
qPCR plate 14	0.046593	0.023119	1139.199	2.015321	0.04410549	<0.0001
qPCR plate 15	-0.00436	0.023591	1161.397	-0.18489	0.853346273	<0.0001
qPCR plate 16	0.017643	0.023437	1151.824	0.752816	0.451714024	<0.0001
qPCR plate 17	0.155652	0.023199	1154.853	6.709526	<0.0001	<0.0001
qPCR plate 18	0.102508	0.023229	1154.692	4.413011	<0.0001	<0.0001
qPCR plate 19	0.052811	0.023273	1131.334	2.269159	0.023446309	<0.0001
qPCR plate 20	0.015264	0.023383	1160.788	0.652755	0.514043213	<0.0001
qPCR plate 21	0.029346	0.023433	1145.858	1.252351	0.210697439	<0.0001
qPCR plate 22	-0.00703	0.022984	1133.92	-0.30599	0.75966473	<0.0001
qPCR plate 23	-0.04198	0.02345	1155.558	-1.79022	0.073681055	<0.0001
qPCR plate 24	-0.01607	0.025524	1153.953	-0.62955	0.529116527	<0.0001
qPCR plate 25	0.032122	0.030063	1128.132	1.068505	0.285521536	<0.0001
qPCR row B	0.11563	0.012884	1136.105	8.974376	<0.0001	<0.0001
qPCR row C	0.067461	0.01279	1125.76	5.274391	<0.0001	<0.0001
qPCR row D	-0.0728	0.012873	1132.07	-5.65538	<0.0001	<0.0001
qPCR row E	-0.00207	0.013454	1141.619	-0.15349	0.878035957	<0.0001
qPCR row F	0.014852	0.012821	1124.566	1.158425	0.246936589	<0.0001
qPCR row G	0.027846	0.012982	1144.541	2.144991	0.032163561	<0.0001
qPCR row H	-0.01398	0.013242	1133.391	-1.05551	0.291416627	<0.0001

Table S6: Calf dataset. Fixed effect estimates of the final model. A quadratic function was fitted to age in days and animal ID was fitted as random effect. Variance components are shown in table 2 of the manuscript.

	Estimate	Std. Error	df	t value	p value for factor level	overall p value for factor
(Intercept)	0.191865	0.098877	46.111	1.940429	0.058456	
Linear component of age in days	-0.60565	0.137759	242.438	-4.39647	1.65E-05	<0.0001
Quadratic component of age in days	0.293872	0.135379	237.3025	2.170735	0.030943	<0.0001
Birth season 2	0.069303	0.032652	36.3774	2.122456	0.040673	0.040673
Birth weight	-0.00501	0.002414	36.64214	-2.07671	0.044897	0.044897
qPCR plate 2	0.063097	0.027462	246.3607	2.297618	0.022422	<0.001
qPCR plate 3	0.075581	0.027529	245.6671	2.74552	0.006488	<0.001
qPCR plate 4	0.067166	0.027162	243.4212	2.47284	0.014088	<0.001
qPCR plate 5	0.143915	0.027615	247.275	5.211416	3.95E-07	<0.001
qPCR plate 6	0.049379	0.031789	262.1415	1.553343	0.121548	<0.001
qPCR row B	-0.05229	0.032564	249.8263	-1.6057	0.109603	0.001
qPCR row C	0.035326	0.031299	242.5322	1.128638	0.260166	0.001
qPCR row D	0.010171	0.031753	245.8092	0.32031	0.749006	0.001
qPCR row E	-0.00395	0.03201	242.3175	-0.12341	0.901888	0.001
qPCR row F	-0.07199	0.032427	246.2654	-2.22009	0.027324	0.001
qPCR row G	0.057835	0.031049	245.8499	1.86269	0.063699	0.001

Table S7: Reasons of culling in the cow dataset.

Reason of culling	Absolute number of cows culled	Relative number of cows (%)
fertility problems	77	31.6
mastitis	35	14.3
lameness	34	13.9
productivity	2	0.8
other	96	39.3

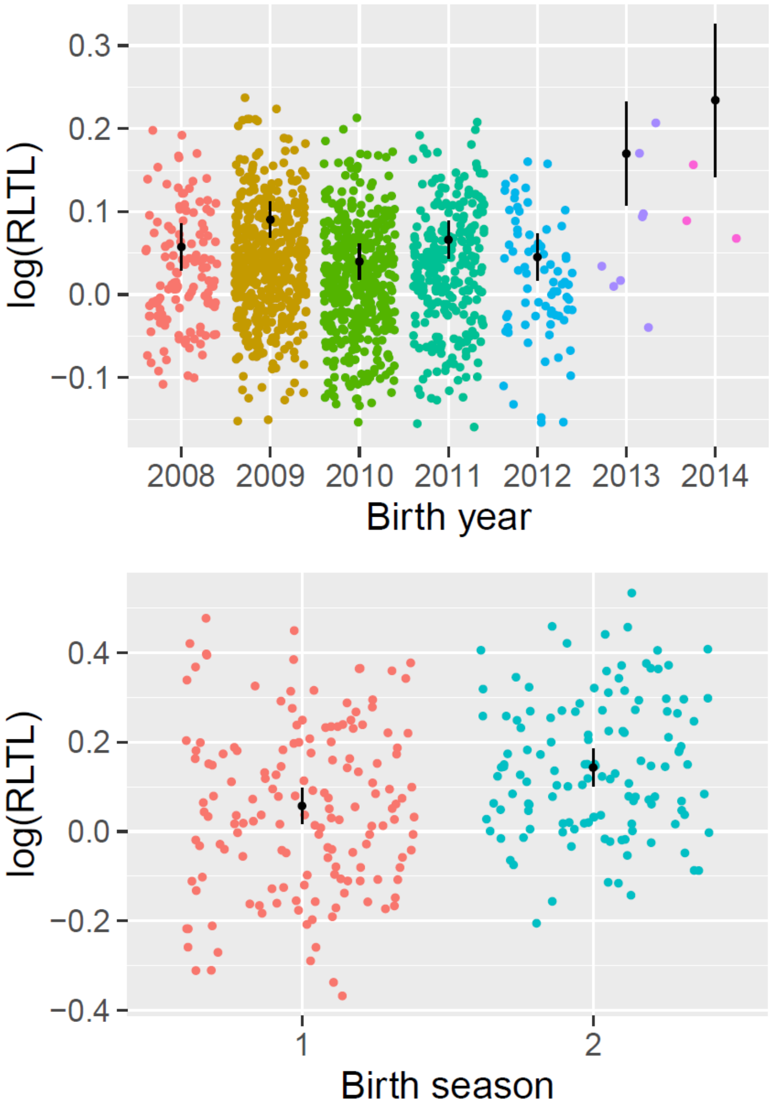

Figure S1: Impact of birth year (cow dataset) and birth season (calf dataset) on RLTL. Model predictions and standard errors are plotted over raw data.

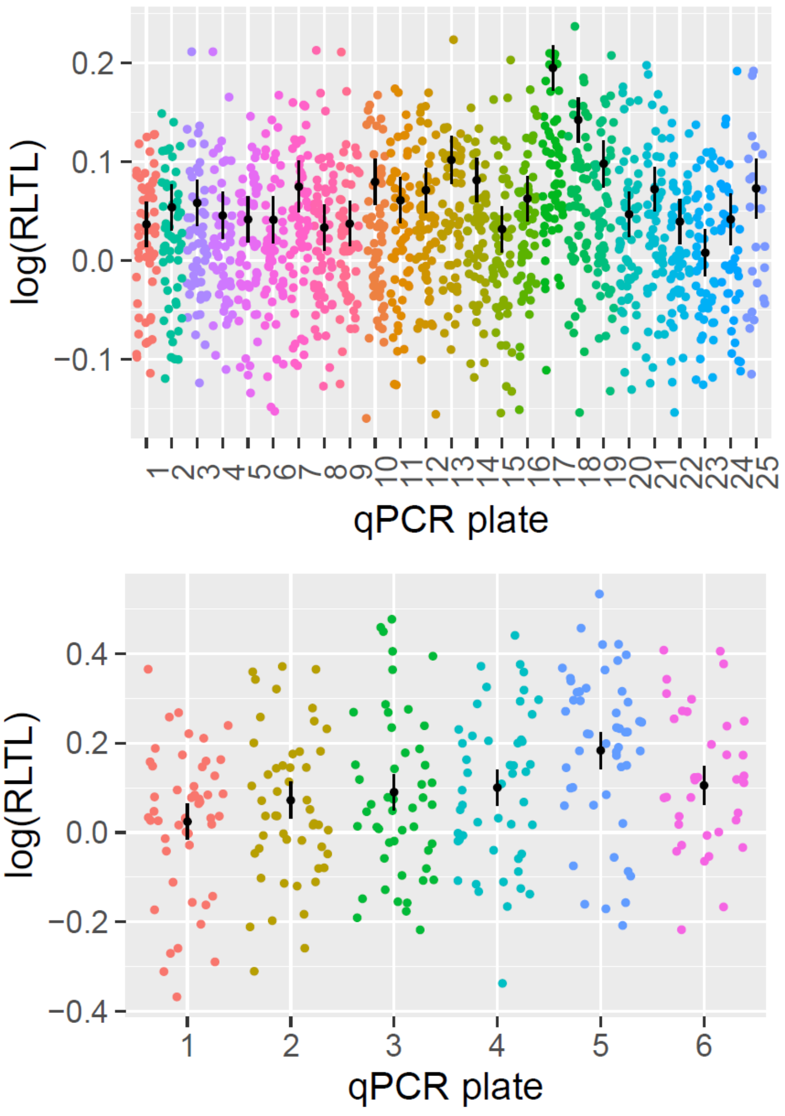

Figure S2: Impact of qPCR plate on RLTL. Model predictions and standard errors are plotted over raw data. Left panel: cow dataset, right panel: calf dataset.

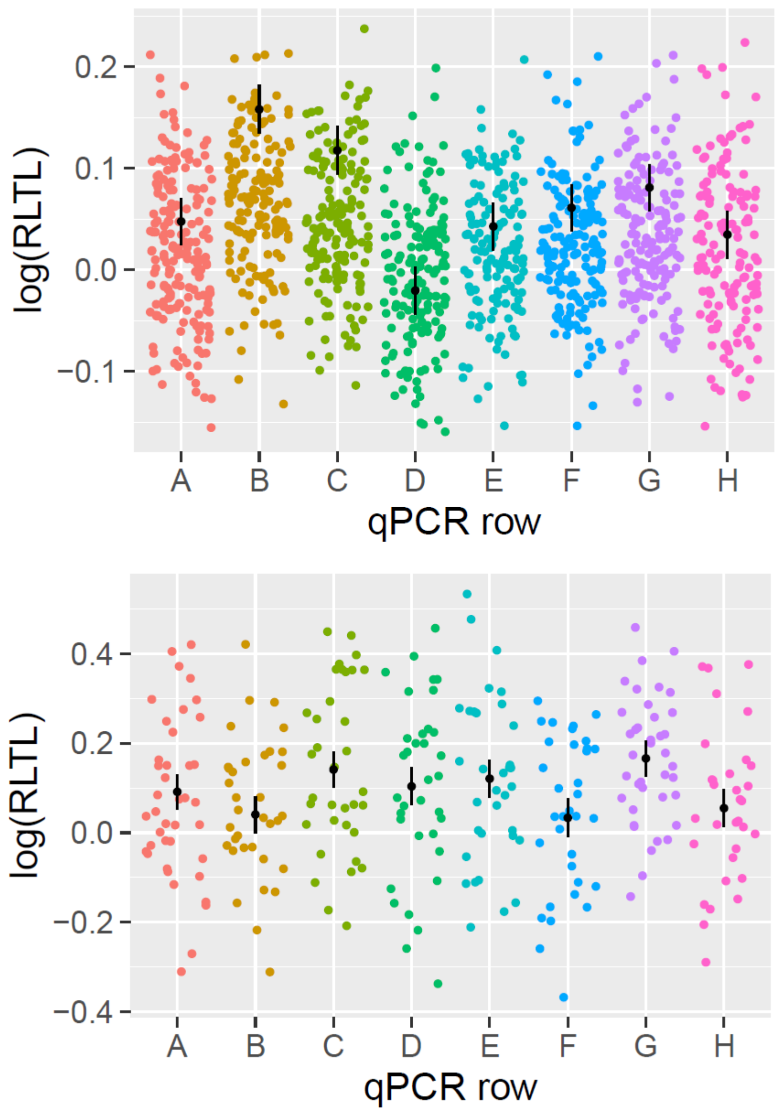

Figure S3: qPCR row effect. Model predictions with standard errors are plotted over raw data. Left panel: cow dataset, right panel: calf dataset.

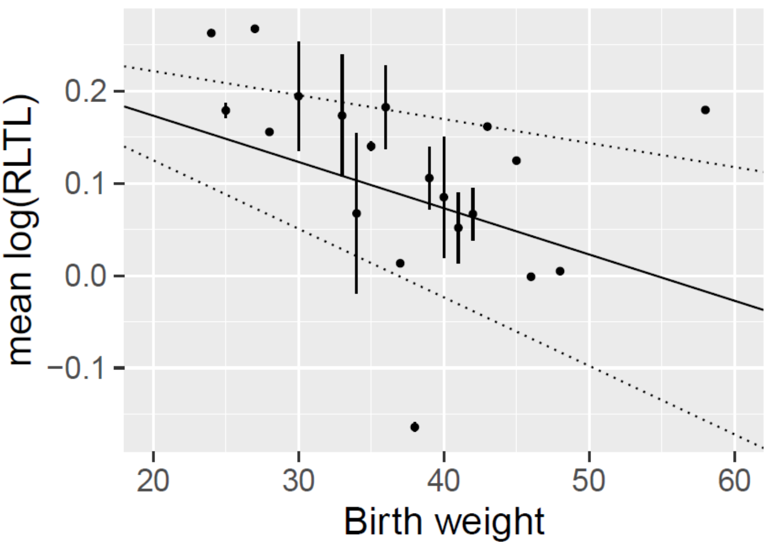

Figure S4: Predicted regression of relative leukocyte telomere length (RLTL) on birth weight. Average raw data with standard errors are shown.
The predicted linear equation was y=-0.005x+0.274 (p=0.045).

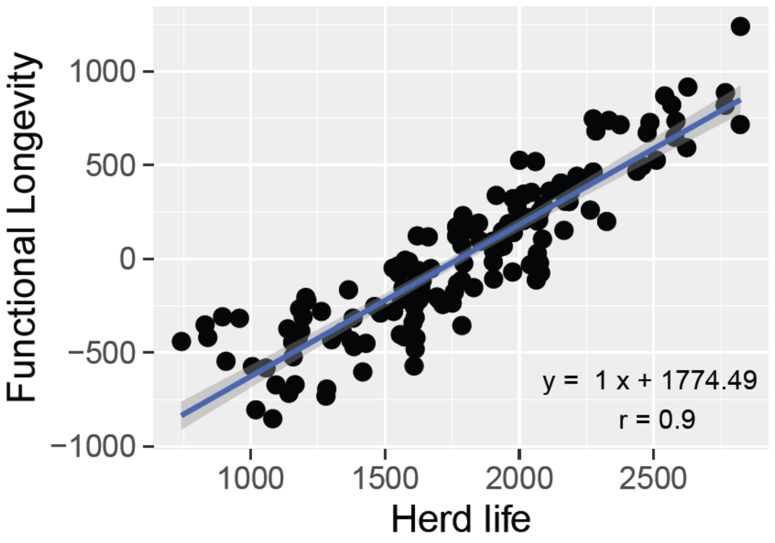

Figure S5: Correlation of functional longevity with productive lifespan (p<0.001).

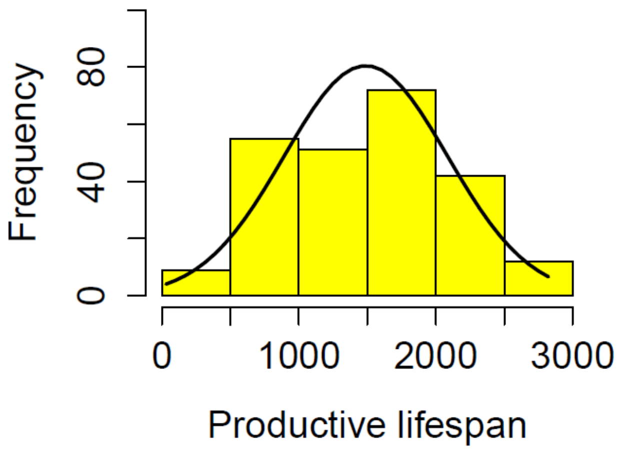

Figure S6: Productive lifespan (=age at culling) in the cow dataset.
